# Cold-Induced RNA-Binding Protein Promotes Glucose Metabolism and Reduces Apoptosis by Increasing AKT Phosphorylation in Mouse Skeletal Muscle Under Acute Cold Exposure

**DOI:** 10.3389/fmolb.2021.685993

**Published:** 2021-07-29

**Authors:** Yang Liu, Peng Liu, Yajie Hu, Yu Cao, Jingjing Lu, Yuying Yang, Hongming Lv, Shuai Lian, Bin Xu, Shize Li

**Affiliations:** National Experimental Teaching Demonstration Center of Animal Medicine Foundation, College of Animal Science and Veterinary Medicine, Heilongjiang Bayi Agricultural University, Daqing, China

**Keywords:** CIRP, glucose metabolism, apoptosis, acute cold exposure, Akt

## Abstract

The main danger of cold stress to animals in cold regions is systemic metabolic changes and protein synthesis inhibition. Cold-induced RNA-binding protein is a cold shock protein that is rapidly up-regulated under cold stimulation in contrast to the inhibition of most proteins and participates in multiple cellular physiological activities by regulating targets. Therefore, this study was carried out to investigate the possible mechanism of CIRP-mediated glucose metabolism regulation and survival promotion in skeletal muscle after acute cold exposure. Skeletal muscle and serum from mice were obtained after 0, 2, 4 and 8 h of acute hypothermia exposure. Subsequently, the changes of CIRP, metabolism and apoptosis were examined. Acute cold exposure increased energy consumption, enhanced glycolysis, increased apoptosis, and up-regulated CIRP and phosphorylation of AKT. In addition, CIRP overexpression in C2C12 mouse myoblasts at each time point under 37°C and 32°C mild hypothermia increased AKT phosphorylation, enhanced glucose metabolism, and reduced apoptosis. CIRP knockdown by siRNA interference significantly reduced the AKT phosphorylation of C2C12 cells. Wortmannin inhibited the AKT phosphorylation of skeletal muscle after acute cold exposure, thereby inhibiting glucose metabolism and aggravating apoptosis. Taken together, acute cold exposure up-regulates CIRP in mouse skeletal muscle, which regulates glucose metabolism and maintains energy balance in skeletal muscle cells through the AKT signaling pathway, thus slowing down the apoptosis of skeletal muscle cells.

## Introduction

A low-temperature environment is a common and unavoidable stressor for humans and livestock in cold regions. Such environments affect neuroendocrine, immune, reproductive, and cardiovascular systems, and cause oxidative stress and apoptosis (Solianik et al., 2014; Cong et al., 2018; Lian et al., 2018). Acute cold exposure cause metabolic disorders, such as increased systemic energy expenditure and increased energy utilization in peripheral tissues (Hao et al., 2015; Yao et al., 2018). Low temperature also has a negative effect on the meat quality and muscle properties (Ferretti, 1992; Short, 2019). Skeletal muscle plays an essential role in the thermogenic metabolic induced by cold exposure due to its ability to produce shivering and non-shivering thermogenesis (Haman, 2006; Brychta and Chen, 2017). Glucose conversion is mostly mediated by skeletal muscle metabolic activation during cold exposure (Blondin et al., 2015), and the maintenance of glucose under cold exposure is of great significance for animal survival (Shibata et al., 1989; Labbé et al., 2015).

When mammals suffer from cold exposure resulting in hypothermia, most protein synthesis is inhibited because the cellular process is usually in cell preservation rather than cell growth (Zhu et al., 2016). However, there are a few proteins that increase in contrast to the general decrease in protein synthesis (Hao et al., 2015), and these include RNA-binding motif protein three and cold-induced RNA-binding protein (CIRP) (Shi et al., 2019; Zhu et al., 2019; Linders et al., 2020). CIRP is a highly conserved RNA binding nucleoprotein with an arginine glycine rich domain and an RNA recognition motif. CIRP mainly regulates the transcription and processing of RNA in the nucleus, and the translation and transformation of mRNA in the cytoplasm (Zhu et al., 2016; Zhong and Huang, 2017). CIRP participates in multiple cell physiological activities through its regulation of targets (Xia et al., 2012), for example, cell survival, proliferation, circadian rhythm regulation, telomere maintenance, tumor formation and development (Zhong and Huang, 2017; Indacochea et al., 2021; Kübler et al., 2021). CIRP expression has been detected in various tissues and cells, especially in mild hypothermia (Liu et al., 2019), hypoxia (Wellmann et al., 2004), UV radiation (Sun et al., 2018), heat stress (Nishiyama et al., 1998), endoplasmic reticulum stress (Khan et al., 2017), and oxidative stress (De Leeuw et al., 2007), where its expression is up-regulated and respond to a variety of stress conditions by changing its expression and regulating mRNA stability through its binding site on the 3′-UTR of its targeted mRNAs (Zhong and Huang, 2017; Zhong et al., 2021). There is interest among researchers with regard to the protective effect of CIRP on the body in cold environments; thus, it is also known as a cold shock protein. CIRP is involved in the physiological regulation of hibernation in amphibians (Sugimoto and Jiang, 2008). CIRP links skin temperature with metabolism and defense mechanisms through AMPK (Higashitsuji et al., 2020). Meanwhile, CIRP plays an irreplaceable role in the cryoprotection and in the treatment of brain injury (Saito et al., 2010; Li et al., 2015; Alkabie and Boileau, 2016). CIRP increase the phosphorylation of protein kinase B (AKT) under mild hypothermia (Li et al., 2015), and AKT signaling can regulate nutrient uptake and metabolism through a variety of downstream targets (Manning and Toker, 2017). In addition, activation of CIRP inhibited apoptosis of neural stem cells during mild hypothermia (Saito et al., 2010).

Therefore, we speculate that CIRP plays a protective role by regulating AKT to participate in glucose metabolism of skeletal muscle under acute cold exposure. Then, this study was carried out to obtain strong evidence to support this conjecture.

## Materials and Methods

### Animals and Acute Cold Exposure

Male ICR mice weighing 30–32 g and aged 8 weeks were selected as experimental animals in this study. The mice were divided into four groups: a normal temperature control group, and 2, 4, and 8 h cold exposure groups (8 mice per group). The mice were first adapted for 7 days in a phytotron, with groups of five mice in standard polystyrene cages. Each cage contained 200 g of soft sawdust bedding, and the bedding was changed twice a week. Mice were free to drink water but food intake was limited (from 8:00 p.m. to 8:00 a.m.). Feeding temperature was set at 28 ± 0.5°C, humidity was 40 ± 5%, illumination intensity was 200 lux, and the light–dark cycle ratio was 12/12 (turn on the lights at 8:00 a.m., turn off the lights at 8:00 p.m.). During the adaptive feeding period, the mice were grabbed for 10 min each day to adapt to capture by the experimenter.

At the end of adaptive feeding, the normal temperature control group remained at 28°C. The cold exposure groups were exposed to 4°C for 2, 4, and 8 h. The mice were anesthetized with ether and euthanized by cervical dislocation. Blood and gastrocnemius muscle samples were collected.

### Biochemical Indices

Fasting blood glucose in mice was determined by a biochemical analyzer with a test strip (IDEXX Laboratories, United States). Insulin and glucagon levels were determined by an ELISA kit (#CEA448Mu and CEB266Mu, Cloud-Clone Corp., United States). Fructose-1,6-diphosphate (FDP) and pyruvic acid (PA) contents in skeletal muscle tissue were determined using assay kits (#BC2240 and BC2200, Solarbio, China).

### Periodic Acid Schiff Staining

The gastrocnemius muscle was fixed with 4% paraformaldehyde. The samples were routinely dehydrated, embedded in paraffin and 5 μm thick sections were prepared. The sections were stained by Periodic Acid Schiff (PAS) stain to examine the glycogen content of gastrocnemius tissues (PAS kit, #G1281, Solarbio, China).

### Western Blotting Analysis

The equivalent protein in gastrocnemius samples from each group was separated by SDS-PAGE, and electrotransferred to a PVDF membrane (#IPVH00010, Millipore, Germany). The PVDF membrane was immersed in 10 ml of Ponceau staining solution and shaken for 5 min. After the clear band appeared, each lane was preserved by Chemidoc XRS Gel Imaging System and analyzed by ImageJ software. Then, the membranes were sealed in the blocking buffer for 1 h at ordinary temperature, and incubated with the primary antibodies overnight at 4°C with shaking. This was followed by incubation with the secondary antibody for 1 h at ordinary temperature. The membranes were visualized by chemiluminescence detection using Luminata Crescendo Western HRP substrate (WBLUR0100, Millipore, Germany). Blots were preserved by the Chemidoc XRS Gel Imaging System and analyzed by ImageJ software.

Antibodies used were as follows: AKT (#90272, 1:1,000), 6-phosphofructo-2-kinase/fructose-2,6-biphosphatase 2 (PFKFB2) (#13045, 1:1,000), phospho-AKT (Ser^473^) (#12694, 1:1,000), phospho-PFKFB2 (Ser^483^) (#13064, 1:1,000), phospho-glycogen synthase kinase-3β (GSK3β) (Ser^9^) (#9323, 1:1,000), glycogen synthase (GS) (#3886, 1:1,000) and phospho-GS (Ser^641^) (#3891, 1:1,000) were purchased from Cell Signaling Technology (United States); glucose transporter-4 (Glut-4) (#ab654, 1:2000), CIRP (#ab166779,1:17,000) and GSK3β (#ab32391, 1:8,000) were purchased from Abcam (United Kingdom); Bcl-2 assaciated x protein (Bax) (#60267-1-lg, 1:2000), B-cell lymphoma-2 (Bcl-2) (#60178-1-lg, 1:2000), Cleaved Caspase-3 (#66470-2-lg, 1:500), and ɑ-tubulin (#11224-1-AP, 1:2000) were purchased from Proteintech (United States). Secondary antibodies were labeled with horseradish peroxidase goat anti-mouse IgG (#SA00001-1, 1:20,000) and goat anti-rabbit IgG (#SA00001-2, 1:20,000), which were also purchased from Proteintech (United States).

### Cell Culture

The mouse myoblast cell line (C2C12) was purchased from Shanghai Cell Bank of the Chinese Academy of Sciences. High glucose DMEM (Gibco, #C11995500BT, United States) containing 10% fetal bovine serum (Gibco, # 10,099, United States), 100 U/mL penicillin and streptomycin (Solarbio, #P1400, China) was used to culture C2C12 cells in a cell incubator at 37°C with 5% CO_2_.

### Cold-Induced RNA-Binding Protein Overexpressed Cell Line Construction and Mild Hypothermia Treatment

The CIRP overexpression lentivirus was obtained from Beijing Hesheng Gene Technology Co., Ltd. (Beijing, China). The virus were diluted with the appropriate multiplicity of infection (200:1) and mixed with polybrene transfection enhancer (Solarbio, #H8761). The final polybrene concentration was 6 μg/ml, and the mixture was added to each well. Cells were again routinely cultured and replaced with conventional medium 24 h later. The efficiency of lentivirus infection was detected by fluorescence microscopy 72 h later. Wild-type cells, empty vector virus-infected cells, and CIRP overexpressed cells were treated with mild hypothermia for 2, 4, and 8 h. The temperature of mild hypothermia treatment was set at 32°C according to our previous body surface temperature measurement and *in vitro* cold exposure model (Liu et al., 2019).

### Cold-Induced RNA-Binding Protein siRNA Interference

CIRP siRNA was obtained from Shanghai Sangon Bioengineering Co., Ltd. (Shanghai, China). All siRNA sequences were shown in Table 1. An appropriate number of cells were seeded on a 6-well plate 24 h before transfection, and transfected when the cell confluence rate reached 70–80%. PBS washed the cells several times after discarding previous cell medium. 1.9 ml pure DMEM medium was added to the 6-well plate. CIRP siRNA and lipofectamine 2000 (#11668030, Invitrogen, United States) were mixed thoroughly with serum-free medium of 50 μL Opti-MEM (Gibco, # 11058021, United States), respectively. After standing for 5 min, the two compounds were fully mixed and stood for 20 min again. Then, 100 μL of the mixture was added to the cells after combining CIRP siRNA and lipofectamine 2000, gently shaken and mixed, and then conventionally cultured. The final content of siRNA was 100 pmol per well, and the final content of Lipofectamine 2000 was 12.5 μL per well. Cells from the cold exposure group after 72 h of transfection were placed in the cell incubator at 32°C and 5% CO_2_ for 4 h, and then total protein was extracted.

**TABLE 1:** CIRP siRNA sequence.

Name	Sense	Antisense
CIRP siRNA#1	5′-GCA​GGU​CUU​CUC​CAA​GUA​UTT-3′	5′-AUA​CUU​GGA​GAA​GAC​CUG​CTT-3′
CIRP siRNA#2	5′-GGC​UAU​GAA​UGG​GAA​GUC​UTT-3′	5′-AGA​CUU​CCC​AUU​CAU​AGC​CTT-3′
CIRP siRNA#3	5′-GGU​CCU​ACA​GAG​ACA​GCU​ATT-3′	5′-AGA​CUU​CCC​AUU​CAU​AGC​CTT-3′
NC siRNA	5′-UUC​UCC​GAA​CGU​GUC​ACG​UTT-3	5′-ACG​UGA​CAC​GUU​CGG​AGA​ATT-3′

### AKT Inhibitor Injection and Cold Exposure Treatment

The following three groups were set up: the control group, DMSO injection group, and wortmannin injection group (8 mice in each group). Wortmannin was dissolved in DMSO and injected intraperitoneally at the dose of 0.5 mg/kg according to the body weight of mice; In the DMSO injection group, DMSO was injected intraperitoneally at the same dose as the negative control. One hour after the injection of wortmannin, the three groups were exposed at 4°C for 4 h. After cold exposure, gastrocnemius muscle samples were collected.

### Statistical Analysis

All data are showed as the mean ± SEM and were analyzed with GraphPad Prism. Comparisons between groups were performed by one-way ANOVA followed by Tukey's post-hoc test. Statistical significance was assumed at *p* < 0.05.

## Results

### Effects of Acute Cold Exposure on Energy Metabolism in Mice

**FIGURE 1:**
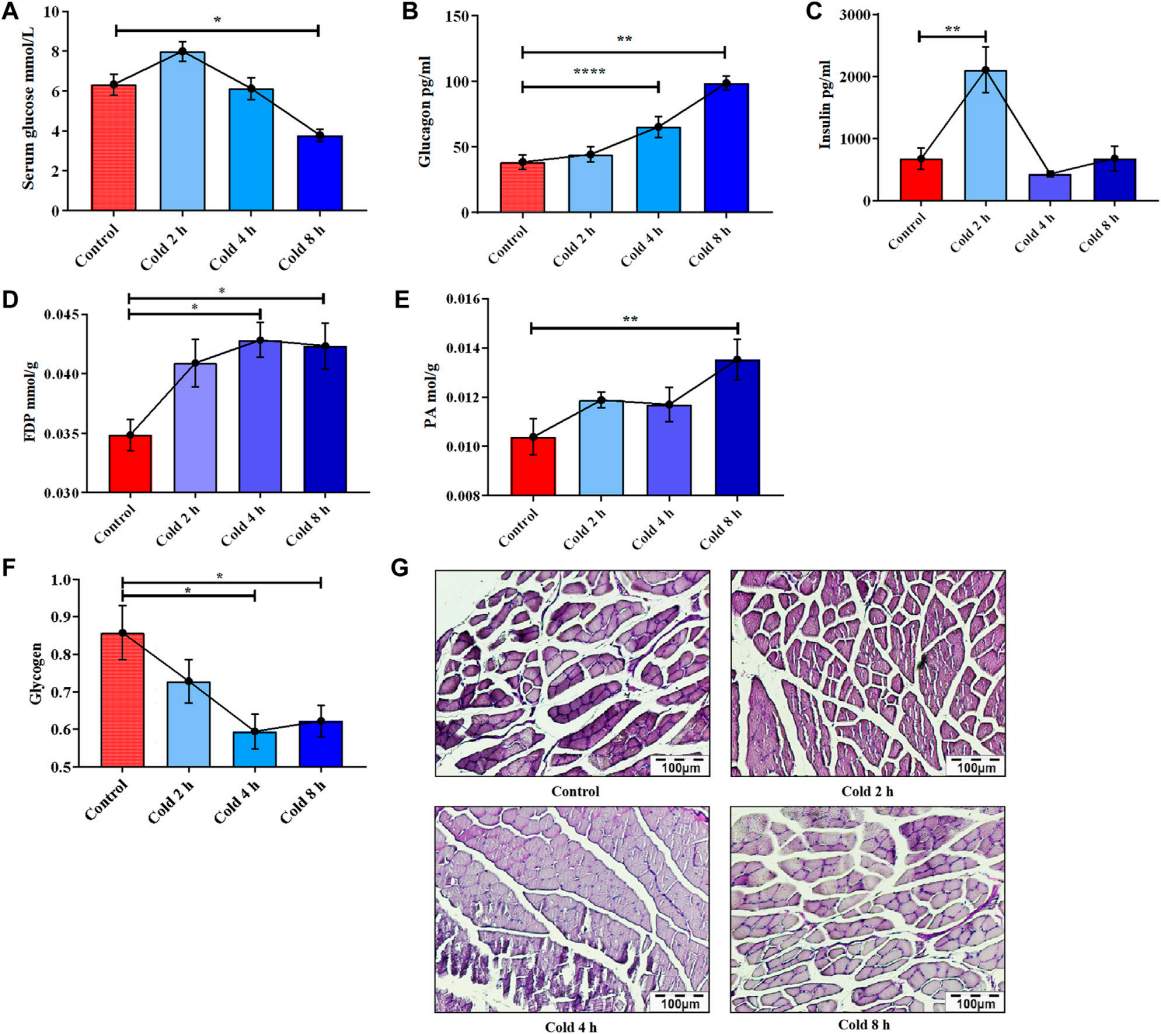
Changes of glucose metabolism induced by acute cold exposure in mice. Effect of acute cold exposure on serum glucose level **(A)**, glucagon level **(B)**, insulin level **(C)** in mice. Effect of acute cold exposure on FDP level **(D)** and PA level **(E)** in the skeletal muscles of mice. Effects of acute cold exposure on glycogen level **(F, G)** in skeletal muscle of mice. The data are presented as the mean ± SEM (n = 8). Statistically significant differences are indicated; *p < 0.05, **p < 0.01. FDP, fructose-1,6-diphosphate; PA, pyruvic acid.

To assess the effect of acute cold exposure on glucose metabolism in mice, serum glucose, insulin, and glucagon levels in all the groups were measured. Serum glucose was significantly decreased after 8 h of cold exposure (*p* < 0.05), although in the cold exposure group, glucose increased slightly at 2 h and decreased at 4 h, but no significant difference was observed (Figure 1A). This small increase may have been the result of active mobilization of the body against cold exposure. In addition, the changes in glucagon and insulin during acute cold exposure were also investigated, as these are antagonistic hormones in regulating blood glucose levels. Glucagon level in the cold exposure group was increased at 4 and 8 h (*p* < 0.01) (Figure 1B). Insulin level fluctuated with prolongation of acute cold exposure (Figure 1C).

It was also found that acute cold exposure enhanced glycolysis of skeletal muscle in mice. This finding is supported by changes in the glycolytic intermediate fructose 1,6-diphosphate (FDP) and the final product PA. FDP content in skeletal muscle significantly changed with acute cold exposure (Figure 1D), as did pyruvic acid (PA) (Figure 1E). PAS staining results demonstrated that the glycogen of skeletal muscle was decreased in a time-dependent manner (Figures 1F,G), suggesting that acute cold exposure caused glycogen depletion in mouse skeletal muscle.

### Acute Cold Exposure Up-Regulated Cold-Induced RNA-Binding Protein, Activated AKT and Enhanced Glucose Metabolism

**FIGURE 2:**
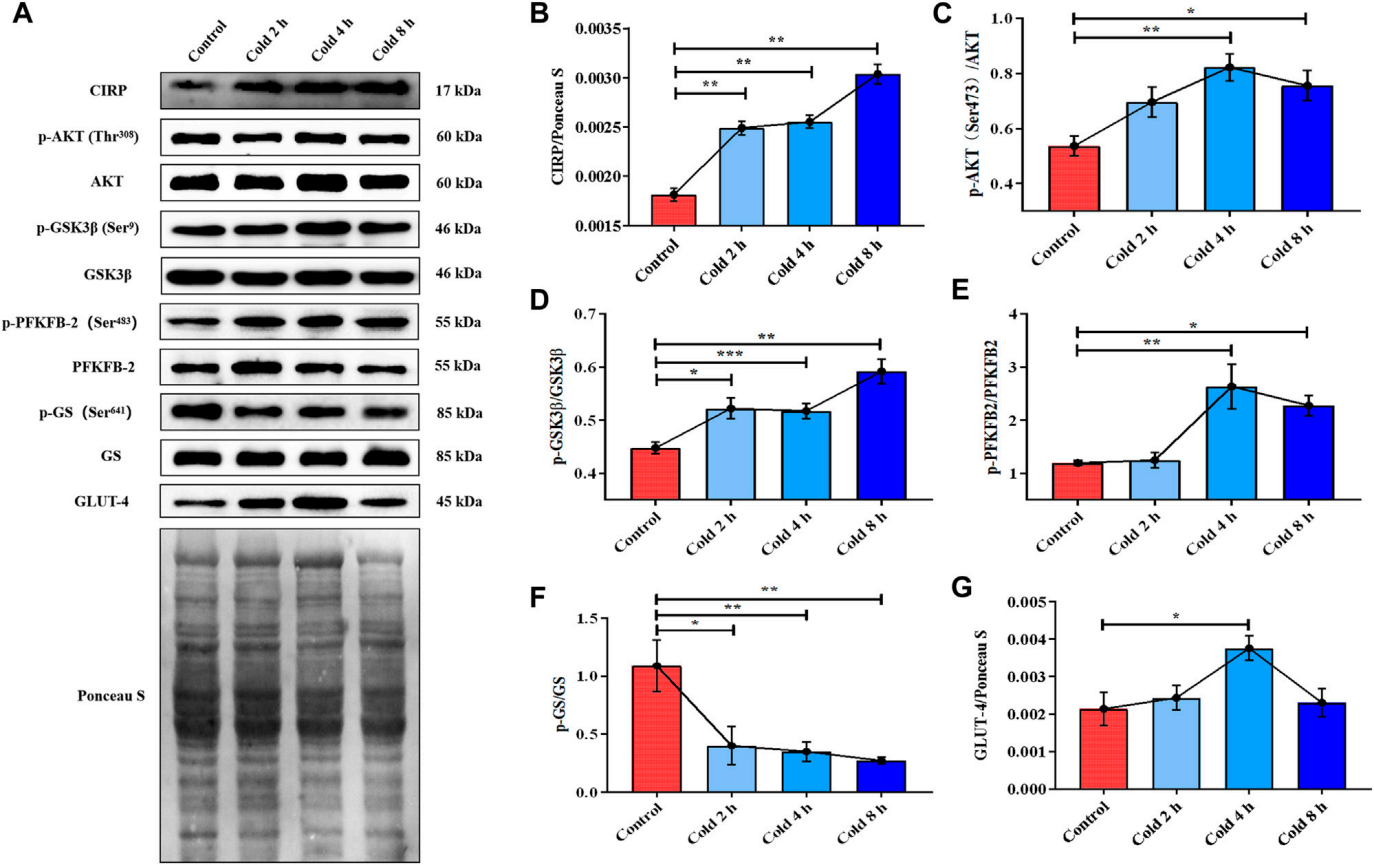
Acute cold exposure up-regulated the expression of CIRP, activated AKT and enhanced glucose metabolism in skeletal muscle of micehanges of glucose metabolism induced by acute cold exposure in mice. **(A)** Western blotting results of glucose metabolism related proteins in mouse skeletal muscle after acute cold exposure. The protein expression levels and phosphorylation status of CIRP **(B)**, AKT **(C)**, GSK3β **(D)**, PFKFB2 **(E)**, GS **(F)** and GLUT-4 **(G)** in the skeletal muscles of mice. The data are presented as the mean ± SEM (n = 8). Statistically significant differences are indicated; *p < 0.05, **p < 0.01. CIRP, cold inducible RNA-binding protein; AKT, protein kinase B; GSK3β, glycogen synthase kinase-3β; PFKFB2, 6-phosphofructo-2-kinase/fructose-2,6- biphosphatase 2; GS, glycogen synthase; GLUT-4, glucose transporter-4.

In order to examine the mechanism of energy metabolism regulation by CIRP under acute cold exposure, CIRP, AKT and glucose metabolism-related proteins were determined. The results showed that CIRP was up-regulated with prolongation of acute cold exposure in a time-dependent manner (Figure 2B). Acute cold exposure subsequently increased AKT phosphorylation at Thr^308^ (Figure 2C). The phosphorylation levels of GSK3β and PFKFB2 were enhanced by acute cold exposure (Figures 2D,E). GLUT4 in the cold exposure group was increased (Figure 2F), but GS and its phosphorylation were inhibited in a time-dependent manner (Figure 2G).

### Acute Cold Exposure Induced Apoptosis in Skeletal Muscle in Mice

**FIGURE 3:**
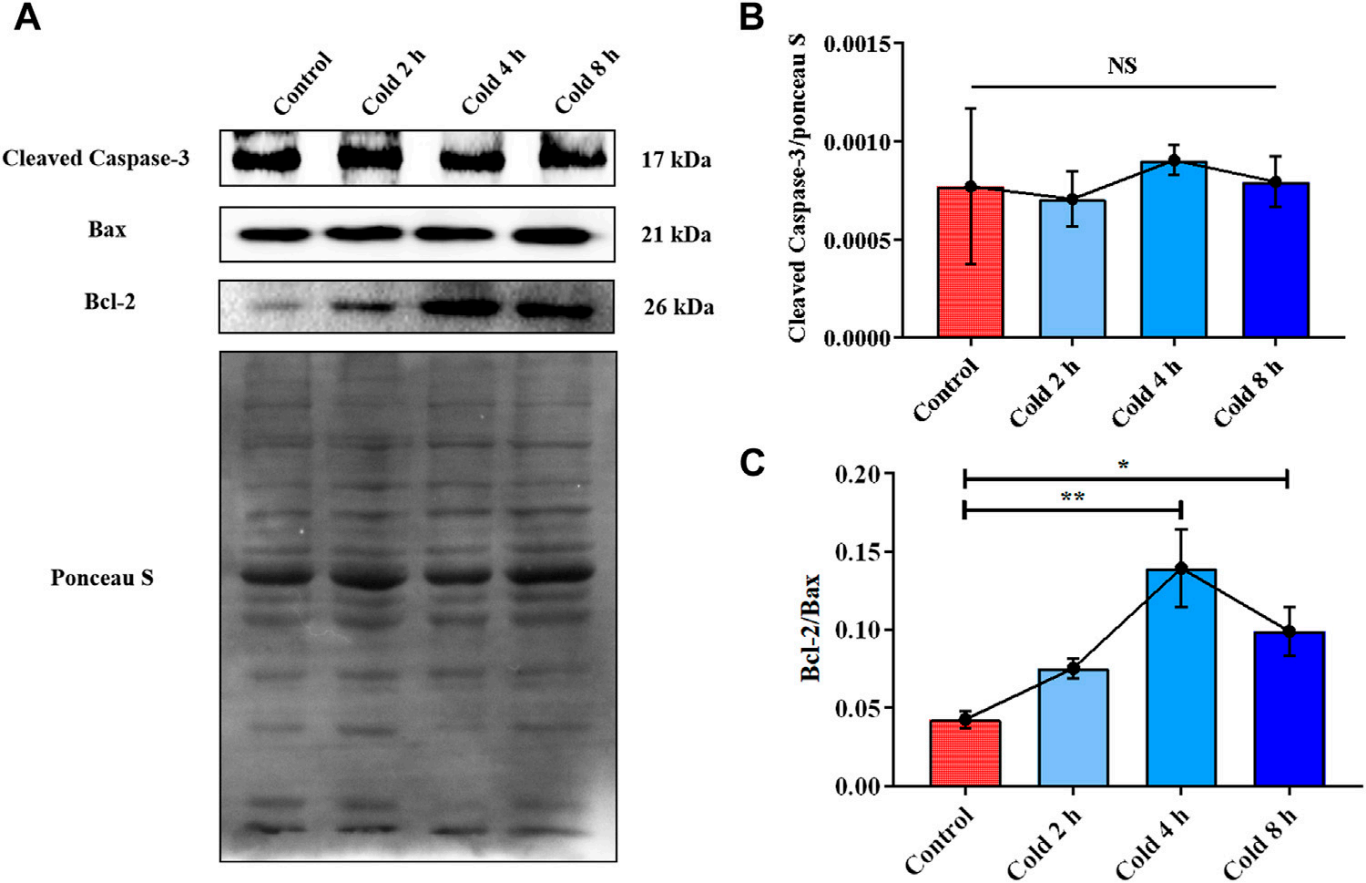
Acute cold exposure induced apoptosis of skeletal muscle in mice. **(A)** Western blotting results of apoptosis related proteins in mouse skeletal muscle after acute cold exposure. The protein expression levels of Cleaved Caspase-3 **(B)**, the expression ratio of Bax and Bcl-2 **(C)** in the skeletal muscles of mice. The data are presented as the mean ± SEM (n = 8). Statistically significant differences are indicated; *p < 0.05, **p < 0.01. Bax, Bcl-2 assaciated x protein; Bcl-2, B-cell lymphoma-2.

In order to examine the effect of acute cold exposure on apoptosis in skeletal muscle, Cleaved Caspase-3, Bax and Bcl-2 in mouse skeletal muscle during acute cold exposure were detected. The Bcl-2 protein family plays an important role in endogenous apoptosis pathway through the interaction of anti-apoptotic and pro-apoptotic proteins within the family (Adams and Cory, 2007). Bcl-2 and Bax belong to anti-apoptotic and pro-apoptotic proteins of the Bcl-2 family, respectively (Peña-Blanco and García-Sáez, 2018). Therefore, the balance between Bcl-2 and Bax can be used as a molecular switch on the apoptotic signaling pathway to determine the direction of cell survival or apoptosis (Leibowitz and Yu, 2010). And the up-regulation of Bcl-2/Bax ratio was used as an indicator of apoptosis inhibition. Cleaved Caspase-3 in the cold exposure group did not change significantly (Figure 3B), while the ratio of Bcl-2 to Bax increased in varying degrees (Figure 3C).

### Overexpression of Cold-Induced RNA-Binding Protein Enhanced AKT Phosphorylation and the Downstream Glucose Metabolism Signaling Pathway Regulated by AKT Under Mild Hypothermia

**FIGURE 4:**
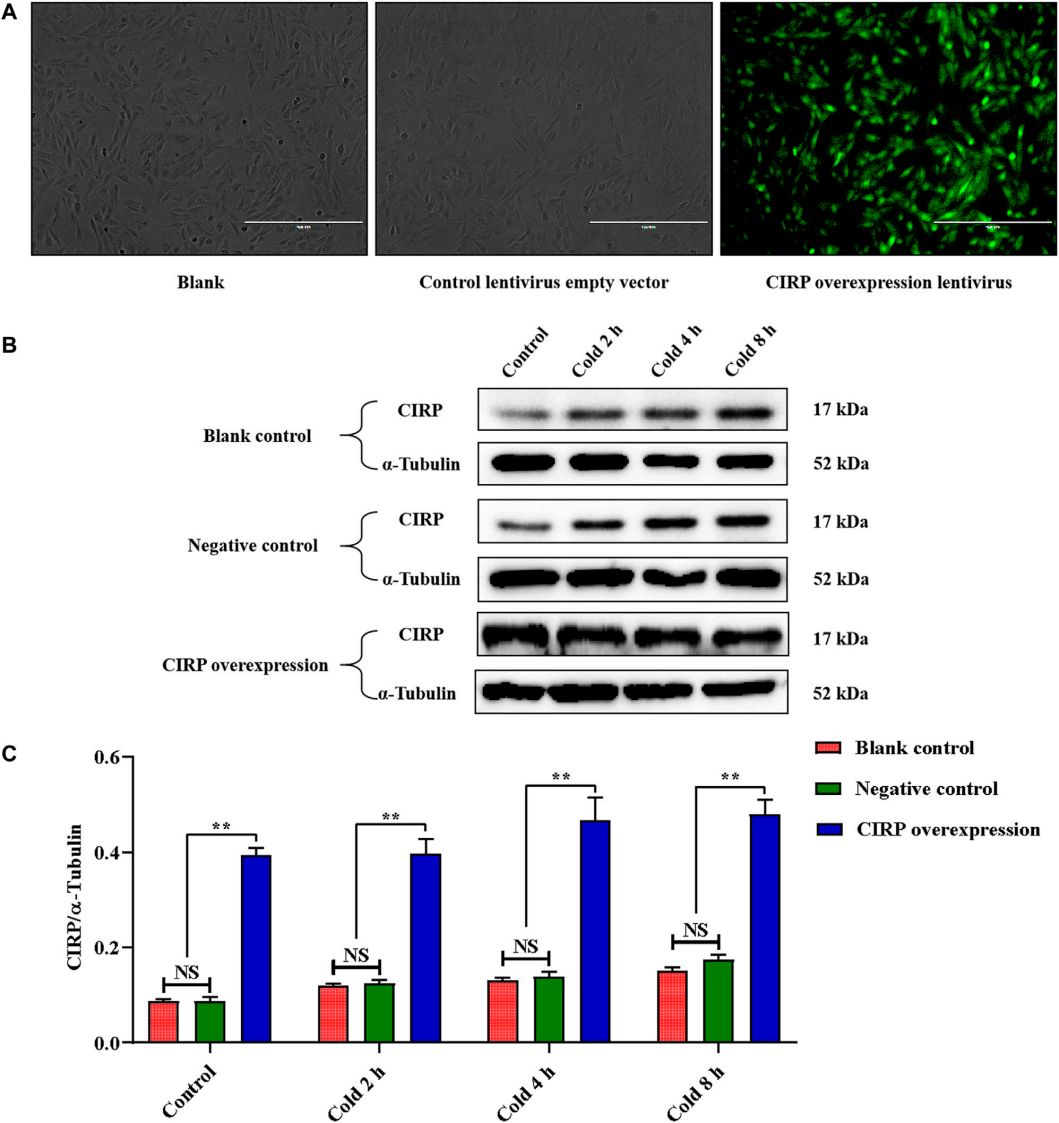
Establishment of CIRP overexpression and its expression changesunder mild hypothermia in C2C12 cells. **(A)** Observation of C2C12 cells infected with CIRP lentivirus for 72 hours by fluorescence microscope. **(B, C)** Effects of mild hypothermia and lentivirus infection on CIRP protein expression in C2C12 cells. The data are presented as the mean ± SEM (n = 8). Statistically significant differences are indicated; *p < 0.05, **p < 0.01. CIRP, cold inducible RNA-binding protein.

In order to further verify our hypothesis, we used the CIRP lentivirus to infect C2C12 mouse myoblasts, and successfully obtained CIRP overexpression *in vitro* (Figures 4A–C). CIRP increased over time in all groups under mild hypothermia at 32°C (Figures 4B,C). When C2C12 cells were infected with CIRP lentivirus, CIRP at the indicated time points was significantly increased (Figures 4B,C).

**FIGURE 5:**
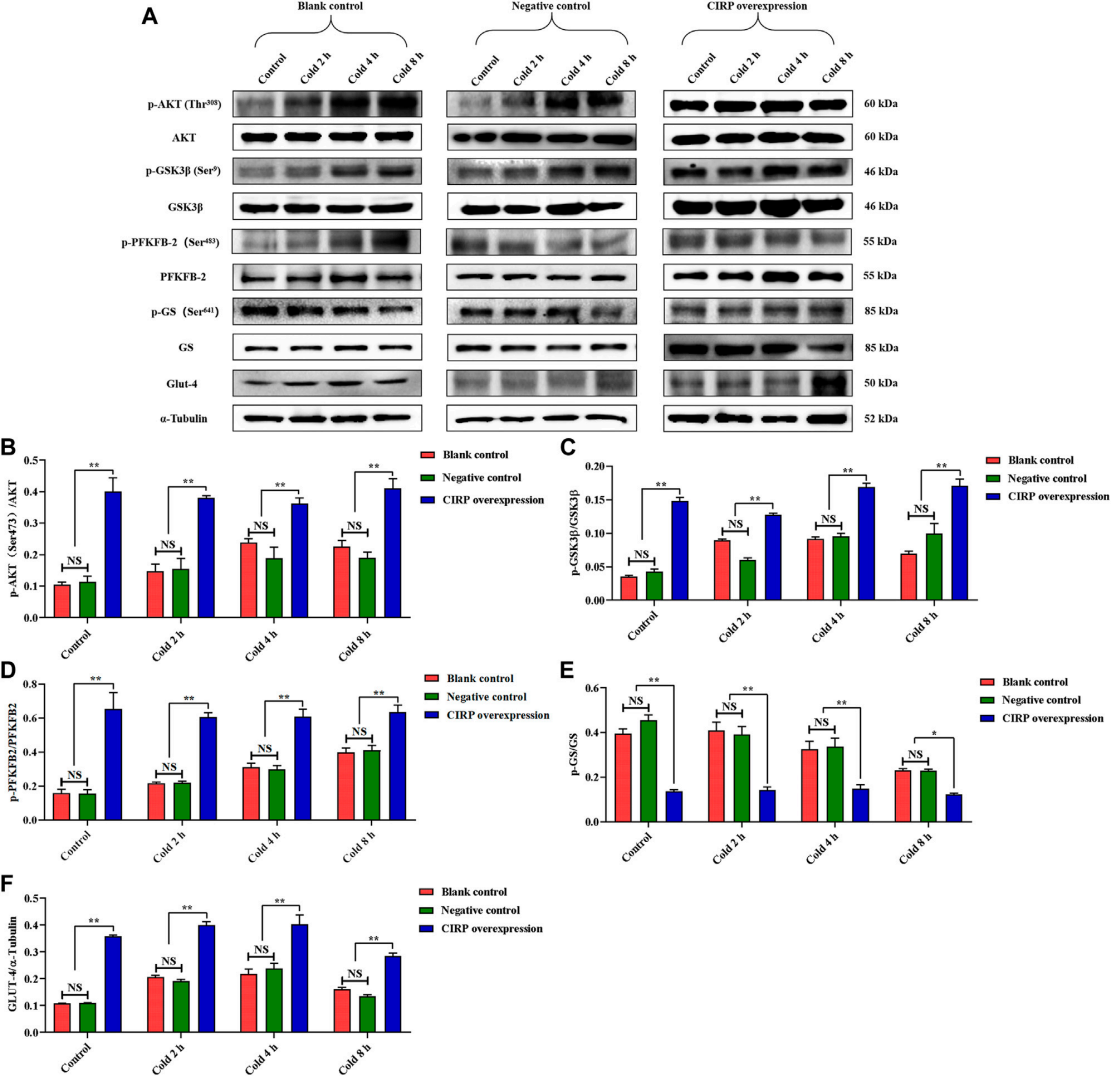
Effects of mild hypothermia and CIRP overexpression on AKT and downstream glucose metabolism pathways. **(A)** Western blotting results of glucose metabolism related proteins in each group. The protein expression levels and phosphorylation status of AKT **(B)**, GSK3β **(C)**, PFKFB2 **(D)**, GS **(E)** and GLUT-4 **(F)** in C2C12 cells. The data are presented as the mean ± SEM (n = 8). Statistically significant differences are indicated; *p < 0.05, **p < 0.01. AKT, protein kinase B; GSK3β, glycogen synthase kinase-3β; PFKFB2, 6-phosphofructo-2-kinase/fructose-2,6-biphosphatase 2; GS, glycogen synthase; GLUT-4, glucose transporter-4.

Subsequently, we detected the expression of the AKT signal pathway and phosphorylation of AKT, GSK3β, PFKFB2 and GS. Mild hypothermia enhanced the phosphorylation levels of AKT, GSK3β and PFKFB2 (Figures 5B–D). The expression level of GLUT4 under mild hypothermia was increased (Figure 5F), but GS and its phosphorylation were inhibited (Figure 5E). More importantly, overexpression of CIRP significantly increased AKT, GSK3β and PFKFB2 and their phosphorylation (Figures 5B–D), as well as the expression of GLUT4 (Figure 5F), while GS and its phosphorylation were inhibited at 37°C and 32°C (Figure 5E).

### Overexpression of Cold-Induced RNA-Binding Protein Attenuates Apoptosis of C2C12 Cells Under Mild Hypothermia

**FIGURE 6:**
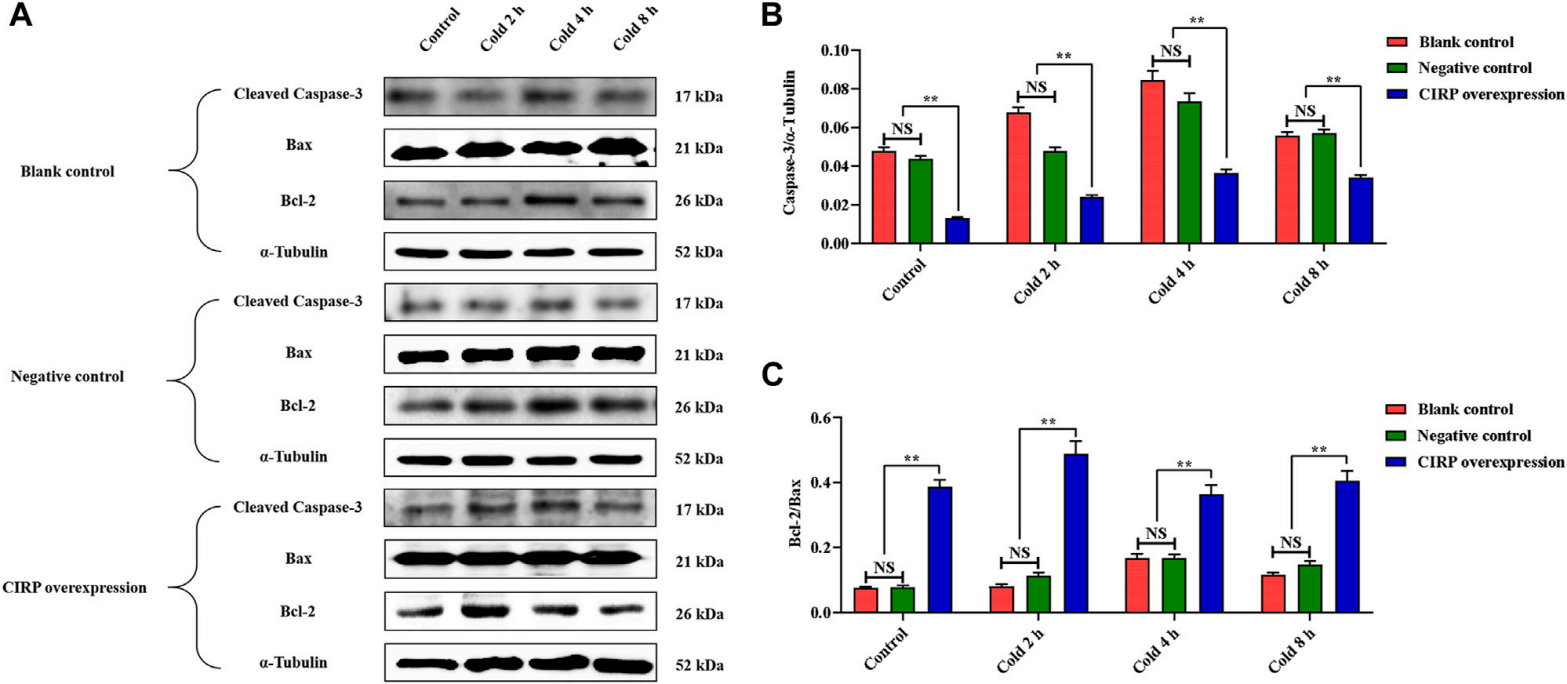
Effects of mild hypothermia and CIRP overexpression on on apoptosis of C2C12 cells. **(A)** Western blotting results of apoptosis related proteins in each group. The protein expression levels of Cleaved Caspase-3 **(B)**, the expression ratio of Bax and Bcl-2 **(C)** in the skeletal muscles of mice. The data are presented as the mean ± SEM (n = 8). Statistically significant differences are indicated; *p < 0.05, **p < 0.01. Bax, Bcl-2 assaciated x protein; Bcl-2, B-cell lymphoma-2.

To evaluate the effects of mild hypothermia and CIRP overexpression on apoptosis of C2C12 cells, the changes of Cleaved Caspase-3, Bcl-2and Bax were analyzed. Mild hypothermia increased Cleaved Caspase-3 (Figure 6B) and the ratio of Bcl-2 to Bax (Figure 6C). Cleaved Caspase-3 was significantly decreased, while the ratio of Bcl-2 to Bax was significantly elevated at 37°C or 32°C (Figures 6B,C).

### Cold-Induced RNA-Binding Protein Knockdown Reduces AKT and Its Phosphorylation Levels

**FIGURE 7:**
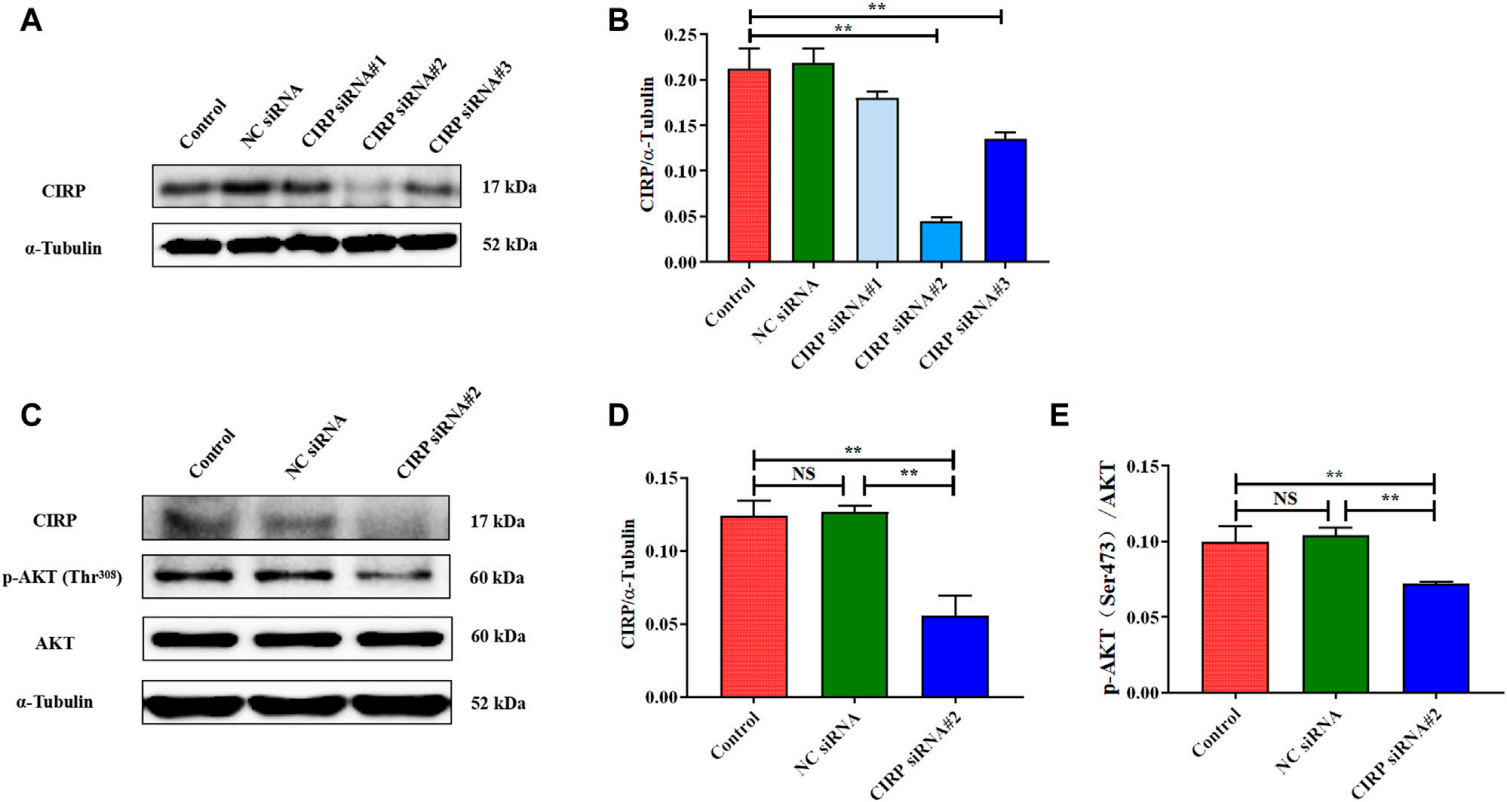
CIRP knockdown reduces AKT and its phosphorylation levels. **(A, B)** Construction and efficiency detection of CIRP siRNA interference. **(C–E)** Effects of CIRP knockdown on AKT and its phosphorylation. The data are presented as the mean ± SEM (n = 8). Statistically significant differences are indicated; *p < 0.05, **p < 0.01. CIRP, cold inducible RNA-binding protein; AKT, protein kinase B.

In order to verify the regulatory effect of CIRP on AKT under mild hypothermia, we first successfully constructed a CIRP knockdown model using CIRP siRNA#2 interference (Figures 7A,B). CIRP protein expression was significantly reduced, leading to a significant decrease in AKT phosphorylation (Figures 7C,D).

### AKT and Its Phosphorylation Are Essential for Promoting Glucose Metabolism and Reducing Apoptosis of Skeletal Muscles in Mice Exposed to Acute Cold Exposure

**FIGURE 8:**
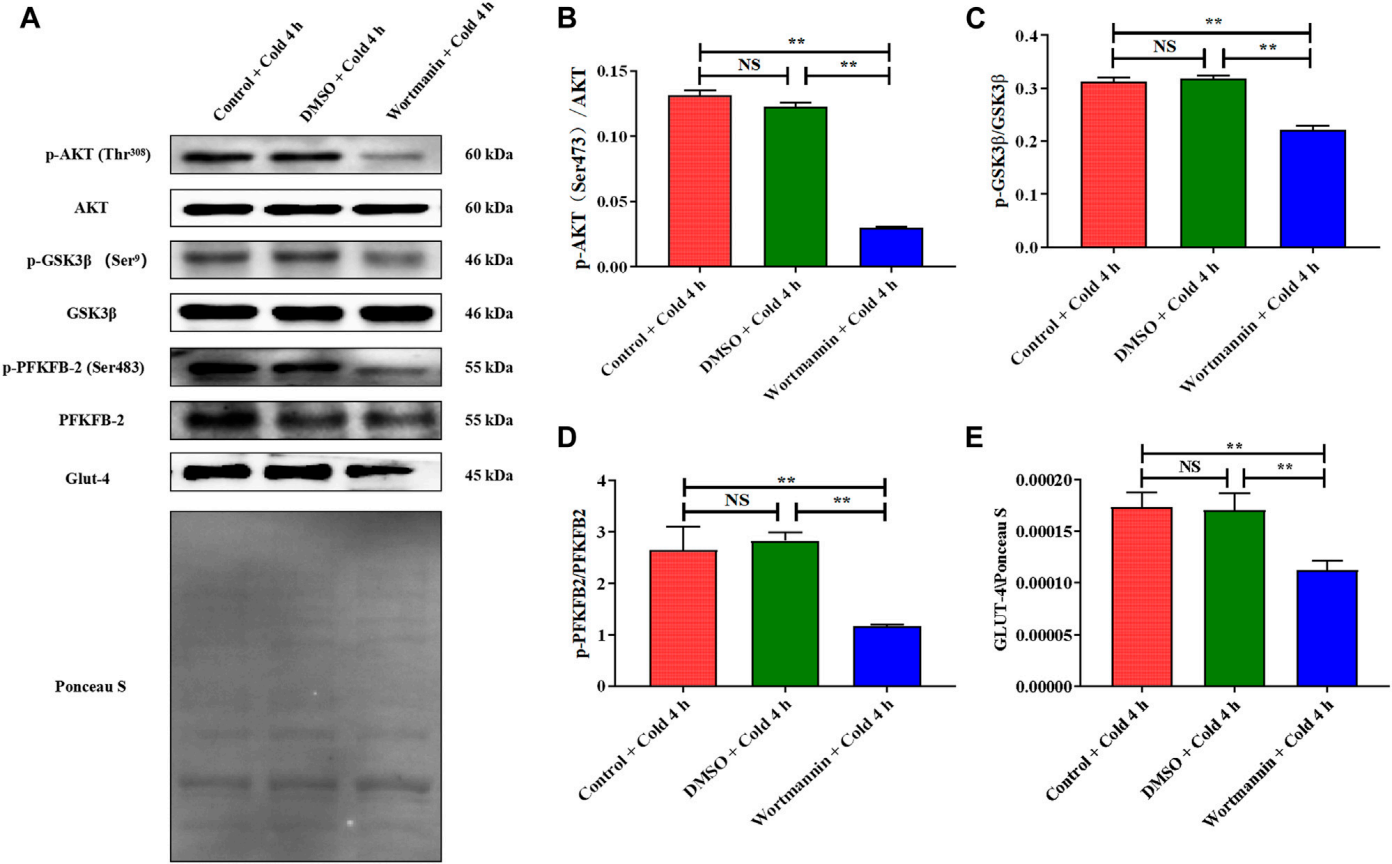
The inhibition of AKT results in the decrease of glucose metabolism in skeletal muscle of mice under acute cold exposure. **(A)** Western blotting results of glucose metabolism related proteins in each group. Effects of wortmannin on protein expression levels and phosphorylation of AKT **(B)**, GSK3β **(C)**, PFKFB2 **(D)** and GLUT-4 **(E)** in skeletal muscle of mice after acute cold exposure. The data are presented as the mean ± SEM (n = 8). Statistically significant differences are indicated; *p < 0.05, **p < 0.01. AKT, protein kinase B; GSK3β, glycogen synthase kinase-3β; PFKFB2, 6-phosphofructo-2-kinase/fructose-2,6- biphosphatase 2; GLUT-4, glucose transporter-4.

To confirm the function of AKT regulated by CIRP in promoting glucose metabolism and reducing apoptosis in skeletal muscle in mice during acute cold exposure, we treated the mice injected with wortmannin (a classic inhibitor of the AKT pathway) under 4 h of acute cold exposure, then determined the expression and phosphorylation levels of AKT and downstream glucose metabolism proteins. The cold exposure for 4 h was due to the more interesting changes in CIRP and AKT in the body under this time node. The AKT phosphorylation was significantly decreased after injection of wortmannin (Figure 8B), which reduced the levels of GSK3β, PFKFB2 and their phosphorylation, and GLUT4 (Figures 8C–E).

**FIGURE 9:**
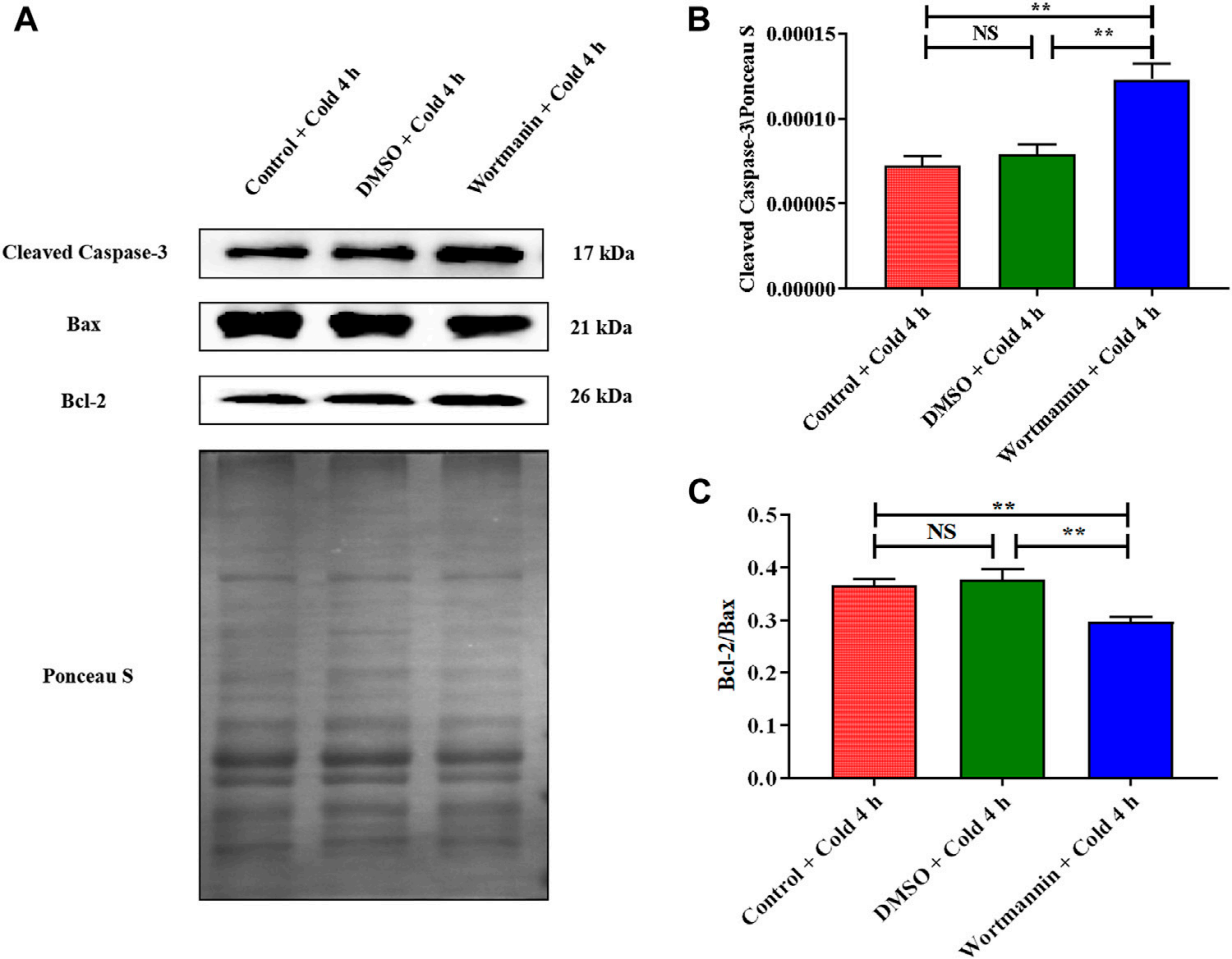
Effect of AKT inhibition on skeletal muscle apoptosis in mice after acute cold exposure. **(A)** Western blotting results of apoptosis related proteins in each group. The protein expression levels of Cleaved Caspase-3 **(B)**, the expression ratio of Bax and Bcl-2 **(C)** in the skeletal muscles of mice. The data are presented as the mean ± SEM (n = 8). Statistically significant differences are indicated; *p < 0.05, **p < 0.01. Bax, Bcl-2 assaciated x protein; Bcl-2, B-cell lymphoma-2.

In addition, Cleaved Caspase-3 was significantly elevated (Figure 9B), and the expression ratio of Bcl-2 to Bax was significantly decreased after wortmannin injection (Figure 9C).

## Discussion

Hypothermia is an environment that imposes high energy demands. Therefore, exposure to a cold environment can cause serious systemic metabolic disorders, such as increased utilization of energy in peripheral tissues, increased systemic energy consumption, and increased glucose production in the liver (Hao et al., 2015; Yao et al., 2018). These changes in energy metabolism are mainly used for cold-induced thermogenesis to maintain the body temperature of thermostatic animals (Brychta and Chen, 2017). Fatty acids are the main energy source in cold-induced thermogenesis, but glucose is essential (Labbé et al., 2015). There is also evidence that glucose is conducive to maintaining an elevated fatty acid oxidation rate during cold exposure (Shibata et al., 1989). The homeostasis of blood glucose level during cold exposure is very important to ensure the energy supply and survival of various tissues (Vallerand et al., 1990; Wang and Wahl, 2014). Results have shown that the blood glucose level decreased significantly in the late stage of acute cold exposure, which further proved that acute cold exposure affected the changes in energy metabolism and the increased utilization and uptake of glucose in peripheral tissues. Our results showed that glucagon levels remained stable at the beginning of acute cold exposure and then increased continuously. However, insulin levels increased at the beginning of acute cold exposure, and then fluctuated. Insulin and glucagon act synergistically to regulate the production and utilization of blood glucose to maintain normal levels (Sharabi et al., 2015). Glucagon can promote glycogen decomposition and gluconeogenesis to meet the body's energy needs in nutritional deficiency (Sharabi et al., 2015). This is the main reason for the continuous increase in glucagon with prolongation of acute cold exposure. One of the bases of glucose homeostasis is the dynamic regulation of insulin secretion according to compensatory nutritional or metabolic changes. The irregular changes in insulin level may be caused by rapid energy mobilization and consumption due to cold stress.

Glucose uptake induced by shivering contraction of skeletal muscle depends mainly on GLUT4 (Huang and Czech, 2007; Richter and Hargreaves, 2013). GLUT4 changed in a sequential manner with prolongation of acute cold exposure time, further indicating that glucose uptake in skeletal muscle increased after cold exposure. We found that glycogen content in skeletal muscle decreased after acute cold exposure, suggesting that acute cold exposure may promote muscle glycogen consumption and lead to a decrease in glycogen content. In addition, maintenance of muscle glycogen levels depends greatly on the supply of blood glucose by glycogen depletion in the liver. Our previous study also found that acute cold exposure caused a significant decrease in liver glycogen, which is another powerful confirmation of muscle glycogen changes (Liu et al., 2019). We then examined the AKT/GSK3β/GS pathway, as the above results also indicated that skeletal muscle demonstrates an increased need for skeletal muscle glycogen synthesis during acute cold exposure. GLUT4 acts as a signal to stimulate AKT (Huang and Czech, 2007). The results showed increased phosphorylation of AKT at Thr^308^ during acute cold exposure. AKT phosphorylation regulates glycogen synthesis through GSK3β/GS pathway (Doble and Woodgett, 2003). AKT activation phosphorylates and inactivates GSK3β, leading to the activation of GS (the rate-limiting enzyme in glycogen synthesis), thereby promoting glycogen synthesis (Henriksen, 2010; Beck-Nielsen, 2012; White et al., 2013). Phosphorylation of AKT and GSK3β in skeletal muscle were increased after acute cold exposure, while the phosphorylation of GS was decreased. Therefore, acute cold exposure activates the main signal pathway of glycogen synthesis in skeletal muscle. The production and destruction of fructose-2, 6-diphosphate is catalyzed by a bifunctional enzyme called PFKFB2. 6-phosphofructokinase-1 is a rate-limiting enzyme for glycolysis, which catalyzes the conversion of fructose-6-phosphate to FDP, and its physiological allosteric activator is fructose-2, 6-diphosphate. Therefore, PFKFB2 is regarded as a key regulator of glycolysis (Baillet et al., 2017). We found that the phosphorylation of PFKFB2 at Ser^483^ was enhanced after acute cold exposure, which indicated that the glycolytic activity in skeletal muscle of mice was enhanced after acute cold exposure. FDP is a metabolic intermediate of glycolysis, which can provide sufficient ATP level, promote the glycolysis process and maintain intracellular calcium balance (Bickler and Kelleher, 1992; Rui, 2014). And the final product of glycolysis is PA. Hence, increased levels of FDP and PA more directly indicate the increase of glycolysis in gastrocnemius muscle of mice after acute cold exposure.

CIRP, as a cold shock protein, is up-regulated and then transferred from the nucleus to the cytoplasm, which responds to cell stress and alleviates stress injury (Yang and Carrier, 2001; De Leeuw et al., 2007). Our previous studies have confirmed that up-regulated CIRP in liver induced by acute cold exposure increases cytoprotection (Liu et al., 2019). Consistent with this, we found that acute cold exposure also up-regulated CIRP in the skeletal muscle of mice and reduced apoptosis of skeletal muscle cells. CIRP participates in multiple signal transduction pathways required for cell function, especially the signal pathways related to apoptosis and proliferation (Zhong and Huang, 2017; Lujan et al., 2018). Cleaved Caspase-3 is the activated form of caspase-3 (Choudhary et al., 2015), and it is also the most important marker for detecting various apoptotic signaling induced apoptosis of cells. We did not observe significant changes in skeletal muscle Cleaved Caspase-3 levels after acute cold exposure. Hypothermia can significantly up-regulate the expression of Bcl-2 in endothelial cells (Sakurai et al., 2013). However, up-regulation of CIRP induced by mild hypothermia inhibits the formation of oxygen free radicals and protects neurons from apoptosis (Li et al., 2015). We also found that acute cold exposure increased Bcl-2, and increased the Bcl-2/Bax ratio. Our evidence shows that CIRP alleviates apoptosis in skeletal muscle tissue during acute cold exposure. In addition, the increase in CIRP in response to acute cold exposure caused similar changes in glucose metabolism and apoptosis in both skeletal muscle and liver of mice (Liu et al., 2019). This not only indicates that CIRP has a general protective effect on regulating glucose metabolism and reducing apoptosis during acute cold exposure, but also suggests that CIRP may cause crosstalk between skeletal muscle and liver to regulate systemic glycogen level. Skeletal muscles secrete a variety of peptides to communicate information with fat, liver, pancreas, bone and brain to respond to environmental/metabolic changes for energy homeostasis (Pedersen and Febbraio, 2012).

Based on the above results, we believe that CIRP promotes glucose metabolism and reduces apoptosis by increasing AKT phosphorylation in the skeletal muscle of mice under mild hypothermia. To clarify the regulatory role of CIRP on the AKT signaling pathway, we subsequently overexpressed and knocked down CIRP in the C2C12 cold exposure model *in vitro*. The C2C12 cold exposure model was successfully constructed based on our previous research (Li et al., 2015; Liu et al., 2019). The results of the C2C12 cold exposure model *in vitro* were basically consistent with those *in vivo*, which showed that CIRP was up-regulated, AKT was activated, glucose metabolism and anti-apoptotic gene protein expression were increased in C2C12 mouse myoblasts under mild hypothermia. CIRP overexpression increased the phosphorylation of AKT, GSK3β, PFKFB2, and GLUT4 protein expression in C2C12 cells at each time point, and inhibited the phosphorylation of GS at 37°C and 32°C. This is basically consistent with the research that CIRP increased the phosphorylation of AKT and activated the AKT signaling pathway (Liu et al., 2015). Furthermore, the phosphorylation of AKT was significantly decreased with CIRP knockdown. This further proves that CIRP regulates AKT expression and activation.

AKT is an essential mediator of phosphoinositide 3-kinase in inhibiting apoptosis and promoting cell survival (Feng and Qiu, 2018). AKT acts as an anti-apoptotic agent by inactivating forkhead transcription factors and blocking mitochondrial release of cytochrome C (Goldbraikh et al., 2020). AKT phosphorylation also inactivates Bad and Caspase-9 (Vescovo et al., 2001; Li et al., 2012). Moreover, the AKT signaling pathway increases Bcl-2 and decreases Bax and Cleaved Caspase-3, thereby attenuating TNF-α-induced apoptosis (Li et al., 2019). On the other hand, GSK3β also promotes apoptosis through a variety of pathways, including p53 acetylation, Bcl-2 degradation, Bax phosphorylation, and/or HSF1 phosphorylation (Doble and Woodgett, 2003; Kockeritz et al., 2006; Kazemi et al., 2010). In addition, inhibition of GSK3β activity through phosphorylation of Ser^9^ residues helps protect against oxidative stress and promotes cold exposure protection (Wang et al., 2016). CIRP overexpression significantly decreased Cleaved Caspase-3 expression in C2C12 mouse myoblasts under mild hypothermia, and significantly improved expression of the Bcl-2/Bax ratio. These results suggest that CIRP may attenuate mild hypothermia-induced apoptosis of C2C12 cells through activation of the AKT signaling pathway.

The regulation of CIRP on AKT is fully demonstrated by the overexpression and knockdown of CIRP. We subsequently confirmed the role of the AKT signaling pathway in glucose metabolism and apoptosis in mouse skeletal muscle by the intraperitoneal injection of wortmannin (a classical AKT inhibitor). The AKT signaling pathway regulates glycogen synthesis and glycolysis in C2C12 cells (Park et al., 2016; Yoshida et al., 2017). Inhibition of AKT decreased the expression of GLUT4 and glucose uptake in C2C12 cells (Wang et al., 2017). The results showed that wortmannin significantly decreased AKT phosphorylation and inhibited AKT signaling pathway activity; the downstream GSK3β phosphorylation level was diminished, and the glycogen synthesis was inactivated in skeletal muscle; the phosphorylation level of PFKFB2 was reduced and the glycolytic activity decreased; GLUT4 expression was inhibited and the ability to absorb glucose was weakened in skeletal muscle. Our evidence indicates that AKT is the main regulatory pathway of glucose metabolism in mouse skeletal muscle during acute cold exposure. In addition, AKT plays a vital role in the regulation of skeletal muscle apoptosis. When inhibited, Cleaved Caspase-3 expression is increased and the level of skeletal muscle cell apoptosis is increased; when activated, Cleaved Caspase-3 expression is decreased, and cell apoptosis is inhibited (Li et al., 2012). Cleaved Caspase-3 expression was elevated and the ratio of Bcl-2 to Bax was reduced in the skeletal muscle of mice during acute cold exposure. The inhibition of AKT by wortmannin can significantly enhance the activity of the apoptotic signaling pathway in mouse skeletal muscle during acute cold exposure. Therefore, we believe that AKT and its phosphorylation are essential for promoting glucose metabolism and thereby reducing apoptosis in the skeletal muscles of mice with acute cold exposure.

## Conclusion

**FIGURE 10:**
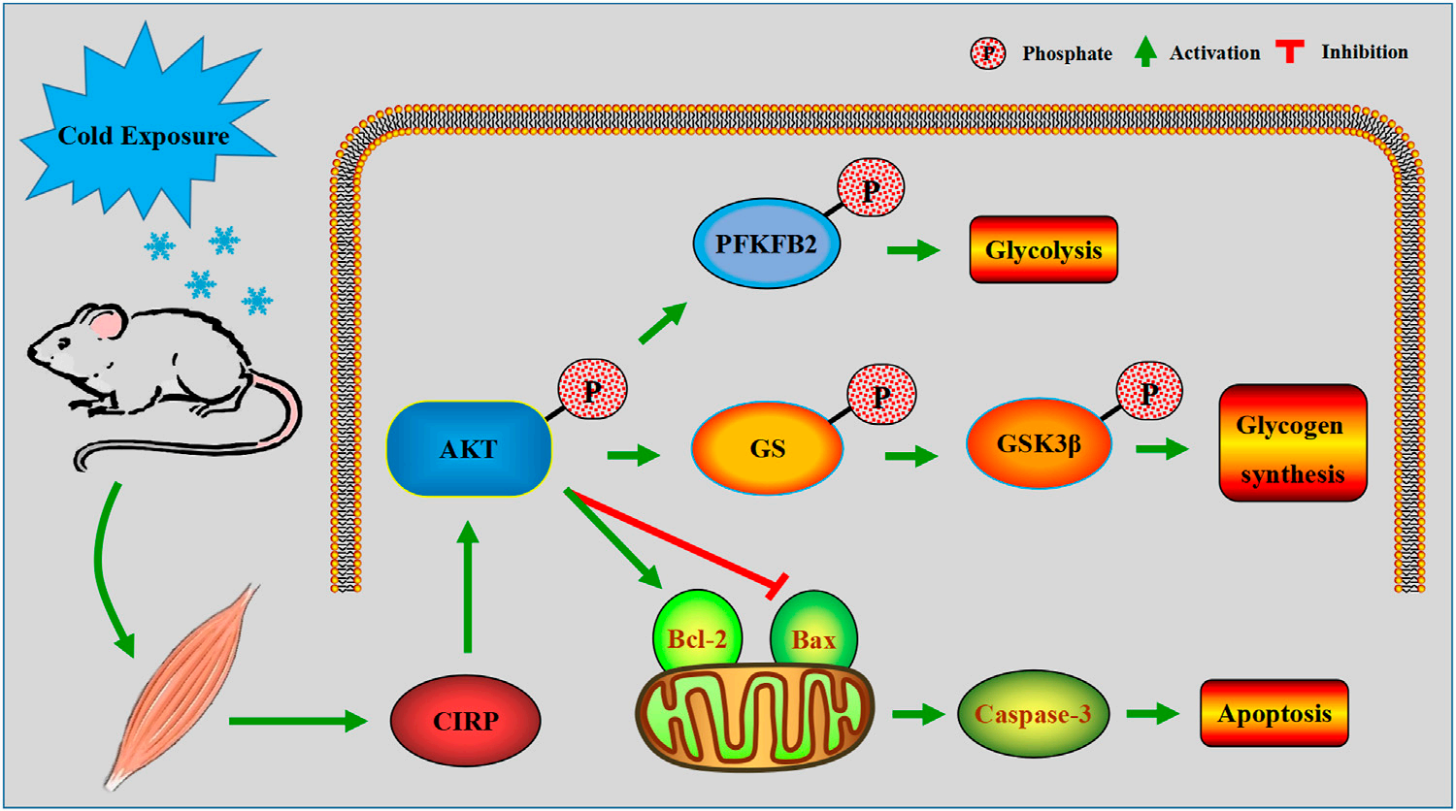
A model of the regulatory mechanism of CIRP in skeletal muscle of mice with acute cold exposure. Arrows indicate promotion, and T-bars indicate inhibition.

The above results demonstrated that up-regulation of CIRP in mouse skeletal muscle due to acute cold exposure can promote glucose metabolism and reduce apoptosis by activating AKT. The proposed role of CIRP in skeletal muscle of mice during acute cold exposure is illustrated in Figure 10.

## Data Availability

The raw data supporting the conclusions of this article will be made available by the authors, without undue reservation.
